# Clinical Specimen-Direct LAMP: A Useful Tool for the Surveillance of *bla*_OXA-23_-Positive Carbapenem-Resistant *Acinetobacter baumannii*

**DOI:** 10.1371/journal.pone.0133204

**Published:** 2015-07-28

**Authors:** Norihisa Yamamoto, Shigeto Hamaguchi, Yukihiro Akeda, Pitak Santanirand, Anusak Kerdsin, Masafumi Seki, Yoshikazu Ishii, Wantana Paveenkittiporn, Robert A. Bonomo, Kazunori Oishi, Kumthorn Malathum, Kazunori Tomono

**Affiliations:** 1 Division of Infection Control and Prevention, Osaka University Graduate School of Medicine, Osaka, Japan; 2 Laboratory of Clinical Research on Infectious Diseases, Research Institute for Microbial Diseases, Osaka University, Osaka, Japan; 3 Faculty of Medicine Ramathibodi Hospital, Mahidol University, Bangkok, Thailand; 4 General Bacteriology Section, National Institute of Health, Department of Medical Sciences, Ministry of Public Health, Bangkok, Thailand; 5 Department of Microbiology and Infectious Diseases, School of Medicine, Faculty of Medicine, Toho University, Tokyo, Japan; 6 Research Service, Louis Stokes Cleveland Veterans Affairs Medical Center, Cleveland, Ohio, United States of America; 7 Infectious Disease Surveillance Center, National Institute of Infectious Diseases, Tokyo, Japan; University Medical Center Groningen, NETHERLANDS

## Abstract

Healthcare-associated infections are a leading cause of morbidity and mortality worldwide. Treatment is increasingly complicated by the escalating incidence of antimicrobial resistance. Among drug-resistant pathogens, carbapenem-resistant *Acinetobacter baumannii* (CRAb) is of increasing concern because of the limited applicable therapies and its expanding global distribution in developed countries and newly industrialized countries. Therefore, a rapid detection method that can be used even in resource-poor countries is urgently required to control this global public health threat. Conventional techniques, such as bacterial culture and polymerase chain reaction (PCR), are insufficient to combat this threat because they are time-consuming and laborious. In this study, we developed a loop-mediated isothermal amplification (LAMP) method for detecting *bla*_OXA-23_-positive CRAb, the most prevalent form of CRAb in Asia, especially in Thailand, and confirmed its efficacy as a surveillance tool in a clinical setting. Clinical samples of sputum and rectal swabs were collected from patients in a hospital in Bangkok and used for LAMP assays. After boiling and centrifugation, the supernatants were used directly in the assay. In parallel, a culture method was used for comparison purposes to evaluate the specificity and sensitivity of LAMP. As a first step, a total of 120 sputum samples were collected. The sensitivity of LAMP was 88.6% (39/44), and its specificity was 92.1% (70/76) using the culture method as the “gold standard”. When surveillance samples including sputum and rectal swabs were analyzed with the LAMP assay, its sensitivity was 100.0%. This method enables the direct analysis of clinical specimens and provides results within 40 minutes of sample collection, making it a useful tool for surveillance even in resource-poor countries.

Healthcare-associated infections are a leading cause of morbidity and mortality worldwide. Treatment is increasingly complicated by the escalating incidence of antimicrobial resistance. Among drug-resistant pathogens, carbapenem-resistant *Acinetobacter baumannii* (CRAb) is of increasing concern because of the limited applicable therapies and its expanding global distribution in developed countries and newly industrialized countries. Therefore, a rapid detection method that can be used even in resource-poor countries is urgently required to control this global public health threat. Conventional techniques, such as bacterial culture and polymerase chain reaction (PCR), are insufficient to combat this threat because they are time-consuming and laborious. In this study, we developed a loop-mediated isothermal amplification (LAMP) method for detecting *bla*_OXA-23_-positive CRAb, the most prevalent form of CRAb in Asia, especially in Thailand, and confirmed its efficacy as a surveillance tool in a clinical setting. Clinical samples of sputum and rectal swabs were collected from patients in a hospital in Bangkok and used for LAMP assays. After boiling and centrifugation, the supernatants were used directly in the assay. In parallel, a culture method was used for comparison purposes to evaluate the specificity and sensitivity of LAMP. As a first step, a total of 120 sputum samples were collected. The sensitivity of LAMP was 88.6% (39/44), and its specificity was 92.1% (70/76) using the culture method as the “gold standard”. When surveillance samples including sputum and rectal swabs were analyzed with the LAMP assay, its sensitivity was 100.0%. This method enables the direct analysis of clinical specimens and provides results within 40 minutes of sample collection, making it a useful tool for surveillance even in resource-poor countries.

## Introduction

*Acinetobacter baumannii* is a gram-negative bacterium that causes nosocomial infections, such as ventilator-associated pneumonia, bacteremia, and urinary tract infections, particularly in immune-compromised patients [1]. This organism has a remarkable capacity to acquire mechanisms that confer resistance to various types of antibiotics [2]. Among these resistant bacteria, carbapenem-resistant *A*. *baumannii* (CRAb) is becoming a major concern in clinical settings because this pathogen is associated with high morbidity and mortality as well as longer hospital stays [3]. Recent studies have reported high rates of resistance worldwide, particularly in Asia-Pacific countries [4]. This issue is especially critical in developing countries where this organism spreads unnoticed and appropriate examinations cannot be performed because of a lack of resources. An easy, rapid, and sensitive detection system is required for proper therapy and isolation precautions [5, 6].

To establish a method that can be widely used in cost-limited situations, we selected LAMP (Loop-mediated isothermal amplification), which is a DNA amplification method that was developed in 2000 [7]. LAMP quickly, specifically, and efficiently amplifies DNA under isothermic conditions. The LAMP method is a valuable tool for the rapid diagnosis of infectious diseases in hospitals [8]. In this study, because CRAb isolates in Asia mostly possess *bla*_OXA-23_ [4, 9–12], we established a novel method for detecting *bla*_OXA-23_-positive CRAb directly from clinical specimens using LAMP (CRAb-LAMP). We also confirmed that this tool could be easily used as a surveillance tool in a hospital: its ease and low cost permit its use in resource-poor countries, and its detection capability for *bla*_OXA-23_ alone is adequate for surveillance.

## Materials and Methods

### Genotyping of CRAb isolates in Thailand

In 2011 clinical isolates of CRAb were collected from various wards and an outpatient department of Ramathibodi Hospital and Phranungklao Hospital in Bangkok in 2011. The sources of specimens were perianal swabs, wounds swabs, tracheal suction and urine, and collected. Duplicate samples were not obtained from one patient. These isolates were analyzed by conventional biochemical testing [13] and MALDI-TOF mass spectrometry (Bruker, Leipzig, Germany) [14] for bacterial identification. The Sensititre (Trek, West Sussex, UK) with the THANF panel of antimicrobial agents was used for the susceptibility tests. The antimicrobial sensitivity testing results (minimum inhibitory concentrations, MICs) were interpreted and reported according to breakpoints published in the CLSI document M100-S23.

The *bla* genes associated with drug resistance were investigated via PCR assays (S1 Table) [11, 12, 15–17]. The PCR mixture (30 μl) contained 0.2 mM of each dNTP, 0.5 μM of each primer, 3 μl of 10× Ex *Taq* buffer, 2.5 U of Ex *Taq* DNA polymerase (TaKaRa Bio Inc., Shiga, Japan), and 1 μl of template DNA that was extracted with a QIAamp DNA mini kit (QIAGEN, Valencia, CA, USA) according to the manufacturer’s protocol. PCR was performed with a thermal cycler (Bio-Rad, Hercules, CA, USA) for 40 cycles. Each cycle consisted of 30 seconds at 95°C, 30 seconds at 55°C, and 45 seconds at 72°C. The amplified products were resolved by agarose gel electrophoresis and visualized with ethidium bromide staining under UV illumination.

### LAMP assay for CRAb isolates

A total of 113 bacterial strains, including *A*. *baumannii*, were used to examine the CRAb-specific reliability of the LAMP reaction, as shown in S2 Table [18–22]. The CRAb strains that were tested included the Thai isolates shown in Table 1, which represent different genotypes, and isolates from Japan, Korea, and Taiwan. We used a diverse collection of clinical strains to evaluate the ability of our LAMP assay to detect various bacterial clones.

**Table 1:** Genotypes of carbapenem-resistant *A*. *baumannii* (CRAb) isolates from hospital patients in Bangkok.

Type number	*OXA -10*	*OXA -23*	*OXA -24*	*OXA -51*	*OXA -58*	*TEM*	*IMP*	*VIM*	*VEB1*	*ARR2*	*CMLA*	Total isolates
1	-	+	-	+	-	-	-	-	-	-	-	1
2	-	+	-	+	-	-	-	-	-	+	-	2
3	+	-	-	+	-	-	-	-	-	-	-	1
4	+	-	-	+	-	-	-	-	-	+	-	1
5	+	-	-	+	-	-	-	-	-	+	+	2
6	+	-	-	+	-	+	-	-	-	-	-	1
7	+	-	-	+	-	+	-	-	-	+	-	1
8	+	-	-	+	-	+	-	-	-	+	+	2
9	+	-	-	+	+	-	-	-	+	+	+	2
10	+	+	-	+	-	-	-	-	-	-	-	6
11	+	+	-	+	-	-	-	-	-	+	-	14
12	+	+	-	+	-	-	-	-	-	+	+	4
13	+	+	-	+	-	-	-	-	+	+	+	2
14	+	+	-	+	-	+	-	-	-	-	-	34
15	+	+	-	+	-	+	-	-	-	+	-	83
16	+	+	-	+	-	+	-	-	-	+	+	16
17	+	+	-	+	+	+	-	-	-	+	-	1
Total^a^	170	163	0	173	3	138	0	0	4	140	28	173
%^b^	98.2	94.2	0	100	1.7	79.8	0	0	2.3	80.9	16.2	100

Total^a^: Total number of isolates with each resistance gene

%^b^: The proportion of isolates with each resistance gene

A set of primers targeting *bla*_OXA-23_ was designed using Primer Explorer V4 software (Net Laboratory, Kanagawa, Japan) based on the genome sequence of *A*. *baumannii* AB0057 (NC_011585), as shown in Table 2 [23]. The primer set targeting the 16S-23S rRNA gene intergenic spacer (ITS) sequence that was used for *A*. *baumannii* identification and as an internal control for the LAMP reaction has been previously described [24]. The reaction mixture (25 μl) consisted of 40 pM each FIP and BIP, 5 pM each F3 and B3, 20 pM each LF and LB, 1.4 mM dNTPs, 10× *Bst* reaction buffer, and 8 U of *Bst* DNA polymerase (Nippon Gene, Osaka, Japan). The mixture was incubated at 60–65°C for 20–60 minutes. The results were evaluated by visual inspection because the LAMP reaction generates insoluble magnesium pyrophosphate as the amplification proceeds [25]. A Loop amp real-time turbidimeter (LA-320; Teramecs, Kyoto, Japan) was also used to confirm the amplification by monitoring the turbidity in the reaction tube in real time according to the OD_650_.

**Table 2:** LAMP primers used for *bla*_oxa-23_ and the ITS sequence.

Target gene		Nucleotide sequence (5′-3′)	Reference
*bla*_OXA-23_	OXA23 F3	GAAGCCATGAAGCTTTCTG	This study
	OXA23 B3	GTATGTGCTAATTGGGAAACA	
	OXA23 FIP	ACCGAAACCAATACGTTTTACTTCTCAGTCCCAGTCTATCAGGA	
	OXA23 BIP	CTGAAATTGGACAGCAGGTTGACTCTACCTCTTGAATAGGCG	
	OXA23 LF	TTTTGCATGAGATCAAGACCGA	
	OXA23 LB	CTGGTTGGTAGGACCATTAAAGGTT	
16S-23S rRNA gene intergenic spacer	ITS-F3	CGGTAATTAGTGTGATCTGAC	[12]
	ITS-B3	CATTTCAGTTTAGAGCACTGT	
	ITS-FIP	TTGCTTAACCTAAACTCTTGAGTGAGAAGACACATTAACTCATTAACAGA	
	ITS-BIP	AGCAAATTAACTGAATCAAGCGTTTACTTAAGCACCGTACAGC	
	ITS-LF	AATTTATTTCAGACTCAATTTTGCCAA	
	ITS-LB	TGGTATGTGAATTTAGATTGAA	

F3: outer forward primer; B3: backward inner primer; LF/LB: loop primersouter backward primer; FIP: forward inner primer; BIP:

### Ethics Statements

Ethical approval for the collection of patient specimens was obtained from the Ethics Committee of Osaka University Graduate School of Medicine and the Ethical Clearance Committee on Human Rights Related to Research Involving Human Subjects Faculty of Medicine Ramathibodi Hospital, Mahidol University. Because sample collection was performed as part of routine clinical work, informed consent was omitted as the both Institutional Review Boards approved. Instead, the patients were informed of the research procedure and wavering rights via a poster in the hospital.

### LAMP assay using clinical samples

All of the clinical samples were collected at the Faculty of Medicine Ramathibodi Hospital, Mahidol University, Bangkok, Thailand from March-June. 2013. Sputum specimens were collected randomly to investigate the efficacy of CRAb-LAMP. As surveillance samples the sputum specimens (intubated patients only) and rectal swabs were obtained in ICUs at the Faculty of Medicine Ramathibodi Hospital

After examination using a conventional culture method, each sample was stored at -20°C until its use in the LAMP assay. Sputazyme (Kyokuto, Tokyo, Japan) was added to 200–500 μl of sputum to reduce the viscosity. The rectal swabs were steeped in 500 μl of saline to dissolve the components. The sputum and rectal swab samples were boiled for 5 minutes and subsequently centrifuged for 10 minutes at 6,600 g. The supernatants were used as templates for LAMP and PCR reaction. Simultaneously, the DNA in the supernatant was extracted using a QIAamp DNA mini kit (QIAGEN, Valencia, CA, USA) according to the manufacturer’s protocol and used for LAMP. The same samples were also used for the PCR assay and to compare those results with the LAMP results.

### Statistical Analysis

Statistical analyses of sensitivity and specificity were analyzed with GraphPad Prism software (GraphPad Software, La Jolla, CA, USA).

## Results

### Genotyping of clinical isolates of CRAb in Thailand

CRAb was mainly manifested by plasmid-encoded beta-lactamases (OXA-23, OXA-24, and OXA-58) categorized as OXA enzymes [1, 9, 26]. This prevalence differed based on the region, and OXA-23-producing strains are highly prevalent in the Asia-Pacific region including Thailand [9–12]. In order to confirm this knowledge and identify a target gene that could be used to detect CRAb via LAMP a total of 173 isolates of CRAb were collected in Bangkok. PCR amplification analyses of the genes associated with carbapenem resistance were performed (Table 1). *bla*_OXA-23_ was identified in most of the isolates (94.2%) as previously described [9–12]. However, *bla*_OXA-24_ and *bla*_OXA-58_, which are frequently reported carbapenemase genes in *A*. *baumannii* samples from Europe and the United States [1, 9, 26], were rarely detected in Thailand. *bla*_OXA-51_ was identified in all of the isolates; however every *A*. *baumannii* strain possesses a chromosomally encoded OXA-51 irrespective of its resistance to carbapenem, and cannot be used in CRAb-detection [9, 27]. Although *bla*_OXA-10_ was found in most of the isolates, OXA-10 was unable to hydrolyze carbapenem and was not an appropriate target for the detection of CRAb [28]. Overall, most CRAb isolates can be detected by amplifying *bla*_OXA-23_; thus, *bla*_OXA-23_ was selected as a LAMP target for the detection of CRAb in this study.

### Establishment of LAMP targeting CRAb carrying *bla*_OXA-23_

**Fig 1:**
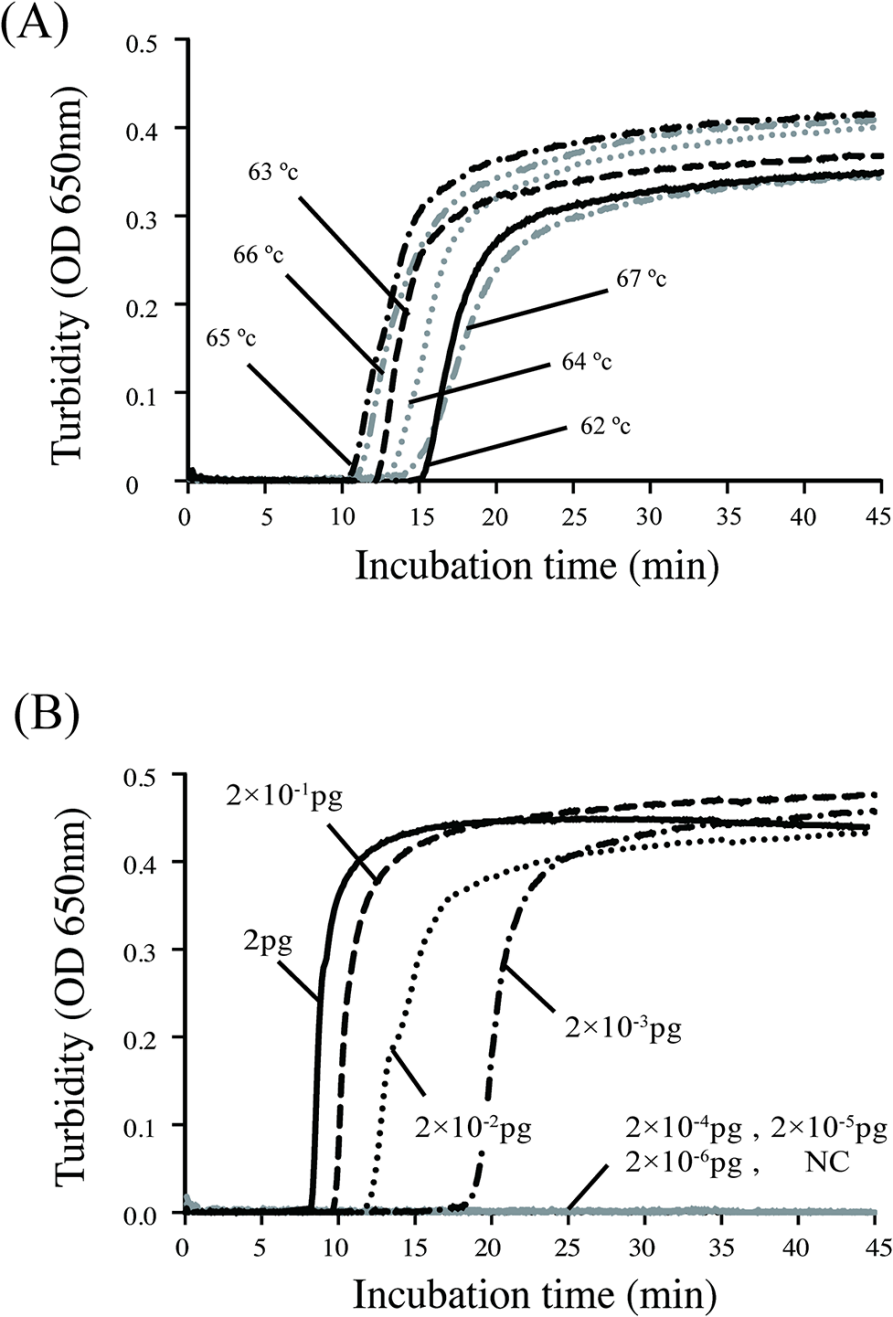
Real-time turbidity assays under various conditions using a turbidimeter. (A) To determine the optimal reaction conditions, a LAMP assay was performed on extracted bacterial DNA at temperatures ranging from 62°C to 67°C. At 65°C, the reaction finished within the shortest period of time, and the negative control remained transparent after 60 minutes of incubation. (B) To determine the detection limit, the extracted DNA templates were serially diluted 10 times (from 2 pg to 2×10^−6^ pg) and used in the LAMP assay. The turbidity was evaluated with a turbidimeter every 5 minutes.

LAMP is becoming increasingly popular as an *in vitro* diagnostic method because it amplifies DNA quickly, efficiently, and highly specifically under isothermic conditions [7, 29]. To identify *A*. *baumannii*, a previously reported assay for the ITS sequence [12] was performed. Because a LAMP assay for *bla*_OXA-23_ has not yet been published, we designed several sets of LAMP primers in advance. To determine the best primer set and the optimal reaction conditions, a LAMP assay was performed on extracted bacterial DNA at temperatures ranging from 62°C to 67°C. The results were evaluated with a turbidimeter, as shown in Fig 1. One successful primer set (Table 2) amplified the target gene, *bla*_OXA-23_, within 20 minutes, and the negative control remained transparent after 60 minutes of incubation. Because the reaction at 65°C produced optimal results (Fig 1), we incubated the samples at 65°C for 20 minutes in all subsequent reactions.

To evaluate the specificity of the LAMP assay for *A*. *baumannii* and CRAb, 113 bacterial strains were used (S2 Table). All of the *A*. *baumannii* isolates were positive for ITS, and all of the CRAb isolates carrying *bla*_OXA-23_ were positive for *bla*_OXA-23_. The results of the LAMP assay matched the PCR results, and LAMP demonstrated 100% sensitivity and 100% specificity for targeting ITS and *bla*_OXA-23_.

To determine the detection limit, the extracted DNA templates were serially diluted 10 times (from 2 pg to 2×10^−6^ pg) and used in the LAMP assay. The turbidity of the LAMP samples was evaluated using a turbidimeter and by visual inspection every 5 minutes (Fig 1). Within 20 minutes, the turbidity of the reaction containing 2×10^−3^ pg of DNA had increased enough for visual inspection; this result indicated that the LAMP assay detected less than 10 CFU per assay as calculated by the amount of DNA extracted from bacterial culture with known CFU. In contrast, conventional PCR can detect 2×10^−1^ pg of DNA using the same templates as the LAMP assay. This result indicated that our LAMP assay was 100 times more sensitive than conventional PCR.

### LAMP assay using clinical samples

We used clinical specimens to examine the feasibility, sensitivity, and specificity of the LAMP assay. To confirm the presence of CRAb in sputum specimens, LAMP assays for ITS and *bla*_OXA-23_ were performed simultaneously, and the samples that were positive for both ITS and *bla*_OXA-23_ were considered CRAb-positive. We refer to this LAMP method as “CRAb-LAMP”. A total of 120 samples were subsequently collected for this study, of which 44 tested positive for CRAb using the culture method. In our CRAb-LAMP assay, 39 samples were considered CRAb-positive with a sensitivity of 88.6% (95% CI: 75.4–96.2%) and a specificity of 92.1% (95% CI: 83.6–97.1%) (Table 3). When sputum specimens were used directly as templates for PCR, no amplicon was obtained due to inhibitory substances present in samples such as blood and sputum [30]. When DNA extracted from the sputum specimens was used as a template for CRAb-LAMP, the sensitivity was 88.6% (95% CI: 75.4–96.2%), the specificity was 90.8% (95% CI: 81.9–96.2%) (Table 3), and the results matched those of the fresh sputum specimens with the exception of one sample (ITS:119/120 and *bla*_OXA-23_:120/120, (accord/total)). This result indicated that LAMP was not affected by interfering substances. When the same DNA samples were directly used in a PCR assay, the sensitivity was 61.3% (95% CI: 45.4–75.6%), and the specificity was 96.0% (95% CI: 73.5–97.9%) for CRAb compared with a culture-based method.

**Table 3:** Sensitivity and specificity of the CRAb-LAMP assay.

			Culture		Sensitivity	Specificity	PPV^a^	NPV^b^	PLR^c^
			Positive	Negative					
Clinical Samples	Sputum Directly	Positive	39	6	88.6	92.1	86.7	94.5	11.2
		Negative	5	70	(75.4–96.2)	(83.6–97.0)	(73.2–95.0)	(85.1–97.8)	
	Extracted DNA	Positive	39	7	88.6	90.8	84.8	93.2	9.63
		Negative	5	69	(75.4–96.2)	(81.4–96.2)	(71.1–93.7)	(84.9–97.8)	

PPV^a^: positive predictive value;NPV^b^: negative predictive value; PLR^c^: positive likelihood ratio

### LAMP assay as a surveillance tool

We next applied surveillance samples to CRAb-LAMP to investigate this method as a surveillance tool. Rectal swab samples were also used as surveillance samples because *A*. *baumannii* colonizes the gastrointestinal tract as well as the oropharynx [31]. After examination using the conventional culture method, each sample was used in a LAMP assay, and the results were compared. If either sample of a patient tested positive, we defined the patient as positive. A total of 108 patients participated in this study, resulting in a sensitivity of 100.0% and a specificity of 83.3% (Table 4). CRAb-LAMP was able to completely identify reservoirs regardless of the type of sample used (S3 Table)

**Table 4:** Sensitivity and specificity of the Surveillance LAMP compared with culture.

		Culture		Sensitivity	Specificity	PPV^a^	NPV^b^	PLR^c^
		Positive	Negative					
LAMP	Positive	30	13	100	83.3	69.7	100	6.00
	Negative	0	65	(88.4–100)	(73.2–90.8)	(53.9–82.8)	(94.5–100)	

PPV^a^: positive predictive value; NPV^b^: negative predictive value; PLR^c^: positive likelihood ratio

## Discussion

*A*. *baumannii* is one of the most problematic pathogens in clinical settings worldwide, primarily because it readily manifests resistance to many different classes of antibiotics [1, 2, 32]. An easy, rapid, and sensitive detection system for CRAb is required. Although classical culture methods have been used to detect CRAb, these require at least 2 days to determine the antibiotic spectrum. Moreover, the sensitivity of the culture method is insufficient to detect patients colonized with CRAb [33]. To solve these problems, we developed “CRAb-LAMP”, which can be used to detect CRAb more easily and rapidly

To identify specific gene candidates, we have genotyped CRAb isolates from Thailand and successfully identified *bla*_OXA-23_. These epidemiological data for CRAb isolates were obtained from just two hospitals; however, the results were similar to previously published data [9–12] and we established LAMP method for *bla*_OXA-23_. Our assay method was not able to detect CRAb in every instance, as a few isolates lacked *bla*_OXA-23_; however, most CRAb isolates can be detected by amplifying *bla*_OXA-23_ due to its high prevalence among CRAb isolates in Thailand [9–12]. The culture-based method was used as the “gold standard”, thus there were some “false-positive” samples; however, these samples may indeed be “true-positives” as a result of the low sensitivity of the culture method [33] and the high sensitivity of the LAMP method. To verify this presumption, we subjected a pair of samples that were obtained from the same patient on different days to both the culture method and CRAb-LAMP. The culture method detected positivity only in the latter sample, whereas LAMP detected positivity in both samples (unpublished data). Therefore, CRAb-LAMP was able to detect CRAb with higher sensitivity.

Three recent reports have described the detection of *A*. *baumannii* via LAMP [16, 34, 35]. None of these studies used clinical samples directly or mentions the utility of LAMP as a surveillance tool. Two of these studies could only detect *A*. *baumannii* and required more time for sample preparation. In clinical settings, clonal spreading is an important concern [1, 36], and we observed that detection of one target gene is sufficient to perform infection control measures. Therefore, our method is expected to be clinically useful in detecting CRAb. The three most popular genotypes of CRAb are *bla*_OXA-23_, which prevails in Asia, and *bla*_OXA-24_ and *bla*_OXA-58_, which are prevalent in Europe and the United States [1, 9, 27]. By designing primers for these target genes, we can detect different genotypes of CRAb easily. The sensitivity and specificity of our CRAb-LAMP method using clinical samples is equivalent to those of currently available diagnostic kits for diseases such as tuberculosis and pathogens such as mycoplasma.

## Conclusion

The CRAb-LAMP assay requires 10 minutes to prepare the template and the reaction master mix, and 20 minutes for the reaction. No special equipment, such as a thermal cycler, is required. The speed of this assay facilitates the early treatment and isolation of CRAb-positive patients. Therefore, our method can facilitate the detection of CRAb and provide a useful tool for hospitals in resource-poor countries, especially in the event of an outbreak.

## Supporting Information

S1 Table PCR primers used for gene identification. To analyze the genotypes of CRAb, the *bla* genes associated with drug resistance were investigated via PCR assays shown in this table.
(DOCX)

S2 Table Bacterial strains used in this study. A total of 113 bacterial strains shown in this table were used to examine the CRAb-specific reliability of the LAMP reaction.
(DOCX)

S3 Table Sensitivity and specificity of the CRAb-LAMP assay. CRAb-LAMP was performed as a surveillance tool. When either of the samples was used, the sensitivity and specificity showed all most the same level regardless of the type of sample used.
(DOCX)
