# Supplementary material for: Clinical Specimen-Direct LAMP: A Useful Tool for the Surveillance of bla OXA-23-Positive Carbapenem-Resistant Acinetobacter baumannii
Source: PLoS One. 2015 Jul 28;10(7):e0133204. doi: 10.1371/journal.pone.0133204 (PMC4517775; doi:10.1371/journal.pone.0133204)
Supplement: S1 Table — To analyze the genotypes of CRAb, the bla genes associated with drug resistance were investigated via PCR assays shown in this table. (DOCX) [file pone.0133204.s001.docx]

**S1 Table. PCR primers used for gene identification**

| Target gene | Description | Primer | Nucleotide sequence (5′-3′) | Reference |
| --- | --- | --- | --- | --- |
| *OXA-10* | Class D β-lactamase gene | OXA-10CASB | TTAGGCCTCGCCGAAGCG | [15] |
|  |  | OXA-10CASF | CTTTGTTTTAGCCACCACCAATGATG |  |
| *OXA-23* | Class D β-lactamase gene | OXA-23F | GATGTGTCATAGTATTCGTCG | [16] |
|  |  | OXA-23R | TCACAACAACTAAAAGCACTG |  |
| *OXA-24* | Class D β-lactamase gene | OXA-24F | GTACTAATCAAAGTTGTGAA | [16] |
|  |  | OXA-24R | TTCCCCTAACATGAATTTGT |  |
| *OXA-51* | Class D β-lactamase gene | OXA-51U | AACAAGCGCTATTTTTATTTCAG | [17] |
|  |  | OXA-51L | CCCATCCCCA ACCACTTT |  |
| *OXA-58* | Class D β-lactamase gene | OXA-58F | ACAGCTTATATTCCTGCATCTACATT | [11] |
|  |  | OXA-58R | GCCTATTTGCATATTGCCATAAC |  |
| *TEM* | Class A β-lactamase gene | TEM1F | ATGAGTATTCAACATTTC | [12] |
|  |  | TEM1R | CTGACATTACCAATGCTTA |  |
| *IMP* | Class B β-lactamase gene | IMPF | TTGCCAGATTTAAAAAT | [11] |
|  |  | IMP003 | ACCAGTTTTGCCTTACCATA |  |
| *VIM* | Class B β-lactamase gene | VIMFW | GTCTACCCGTCCAATGGTCTCA | [11] |
|  |  | VIMRV | AGCAAGTCTAGACCGCCCG |  |
| *VEB-1* | Extended spectrum β-lactamase gene | VEB1F | CCACTTCCATTTCCCGATGC | [12] |
|  |  | VEB1R | GGACTCTGCAACAAATACGC |  |
| *ARR2* | Rifampin ADP-ribosyltransferase gene | ARR2F | CATTTCGAGGACGGTCGTAT | [12] |
|  |  | ARR2R | GCCTATTGCGCATAAAATGG |  |
| *CMLA* | Sulfonamide resistance-encoding gene | CMLAF2 | ACTAATGATGGCAGGCAAG | [12] |
|  |  | CMLAR2 | AAGACAGACCGAGCACGACT |  |
