# Supplementary material for: Clinical Specimen-Direct LAMP: A Useful Tool for the Surveillance of bla OXA-23-Positive Carbapenem-Resistant Acinetobacter baumannii
Source: PLoS One. 2015 Jul 28;10(7):e0133204. doi: 10.1371/journal.pone.0133204 (PMC4517775; doi:10.1371/journal.pone.0133204)
Supplement: S2 Table — A total of 113 bacterial strains shown in this table were used to examine the CRAb-specific reliability of the LAMP reaction. (DOCX) [file pone.0133204.s002.docx]

**S2 Table . Bacterial strains used in this study**

| Species | No. of strains | Strain number | Reference |
| --- | --- | --- | --- |
| *Acinetobacter baumannii*  (drug susceptible) | 39 | Isolate No. 10634-10640,  clinical isolates | [18] |
| *Acinetobacter baumannii*  (*bla*_OXA-23_-positive CRAb) | 74 | Isolate No. 10629-10633, 10641,  clinical isolates | [18] |
| *A. pittii* | 3 | Isolate No. 10643-10645 | [18] |
| *A. calcoaceticus* | 4 | Isolate No. 10646-10649 | [18] |
| *A. nosocomialis* | 4 | Isolate No. 10650-10653 | [18] |
| *A. lwoffii* | 1 | Isolate No. 10654 | [18] |
| *Haemophilus influenzae* | 2 | GB3291, ATCC10211 | [19, 20] |
| *Streptococcus pneumoniae* | 1 | D39 | [21] |
| *Staphylococcus aureus*  (MRSA) | 1 | NCTC10442 | [22] |
