# Supplementary material for: Clinical Specimen-Direct LAMP: A Useful Tool for the Surveillance of bla OXA-23-Positive Carbapenem-Resistant Acinetobacter baumannii
Source: PLoS One. 2015 Jul 28;10(7):e0133204. doi: 10.1371/journal.pone.0133204 (PMC4517775; doi:10.1371/journal.pone.0133204)
Supplement: S3 Table — CRAb-LAMP was performed as a surveillance tool. When either of the samples was used, the sensitivity and specificity showed all most the same level regardless of the type of sample used. (DOCX) [file pone.0133204.s003.docx]

**S3 Table. Sensitivity and specificity of the CRAb-LAMP assay.**

|  | | | Culture | | Sensitivity | Specificity | PPV^a^ | NPV^b^ | PLR^c^ |  |
| --- | --- | --- | --- | --- | --- | --- | --- | --- | --- | --- |
|  | | | Positive | Negative |  |  |  |  |  |  |
| Surveillance  Samples | Sputum | Positive | 20 | 9 | 100 | 79.6 | 69.0 | 100 | 4.89 | |
|  |  | Negative | 0 | 35 | (83.1-100) | (64.7-90.2) | (49.2-84.7) | (90.0-100) |  |  |
|  | Rectal  Swab | Positive | 13 | 26 | 100 | 72.6 | 33.3 | 100 | 3.65 | |
|  |  | Negative | 0 | 69 | (75.3-100) | (65.2-81.2) | (19.9-50.2) | (94.8-100) |  |  |

PPV^a^: positive predictive value; NPV^b^: negative predictive value; PLR^c^: positive likelihood ratio
